# The landscape of the immunoglobulin repertoire in endemic pemphigus foliaceus

**DOI:** 10.3389/fimmu.2023.1189251

**Published:** 2023-07-28

**Authors:** Verónica Calonga-Solís, Michael Olbrich, Fabian Ott, Gabriel Adelman Cipolla, Danielle Malheiros, Axel Künstner, Ticiana D.J. Farias, Carolina M. Camargo, Maria Luiza Petzl-Erler, Hauke Busch, Anke Fähnrich, Danillo G. Augusto

**Affiliations:** ^1^ Programa de Pós-Graduação em Genética, Universidade Federal do Paraná, Curitiba, Brazil; ^2^ Medical Systems Biology Group, Lübeck Institute of Experimental Dermatology, University of Lübeck, Lübeck, Germany; ^3^ Department of Biological Sciences, The University of North Carolina at Charlotte, Charlotte, NC, United States

**Keywords:** autoimmunity, immunoglobulin repertoire, environmental factors, B cells, skin disease, pemphigus foliaceus

## Abstract

**Introduction:**

Primarily driven by autoreactive B cells, pemphigus foliaceus (PF) is an uncommon autoimmune blistering skin disease of sporadic occurrence worldwide. However, PF reaches a prevalence of 3% in the endemic areas of Brazil, the highest ever registered for any autoimmune disease, which indicates environmental factors influencing the immune response in susceptible individuals. We aimed to provide insights into the immune repertoire of patients with PF living in the endemic region of the disease, compared to healthy individuals from the endemic region and a non-endemic area.

**Methods:**

We characterized the B-cell repertoire in i) nontreated patients (n=5); ii) patients under immunosuppressive treatment (n=5); iii) patients in remission without treatment (n=6); and two control groups iv) from the endemic (n=6) and v) non-endemic areas in Brazil (n=4). We used total RNA extracted from peripheral blood mononuclear cells and performed a comprehensive characterization of the variable region of immunoglobulin heavy chain (IGH) in IgG and IgM using next-generation sequencing.

**Results:**

Compared to individuals from a different area, we observed remarkably lower clonotype diversity in the B-cell immune repertoire of patients and controls from the endemic area (*p* < 0.02), suggesting that the immune repertoire in the endemic area is under geographically specific and intense environmental pressure. Moreover, we observed longer CDR3 sequences in patients, and we identified differential disease-specific usage of IGHV segments, including increased IGHV3-30 and decreased IGHV3-23 in patients with active disease (*p* < 0.04). Finally, our robust network analysis discovered clusters of CDR3 sequences uniquely observed in patients with PF.

**Discussion:**

Our results indicate that environmental factors, in addition to disease state, impact the characteristics of the repertoire. Our findings can be applied to further investigation of the environmental factors that trigger pemphigus and expand the knowledge for identifying new targeted and more effective therapies.

## Introduction

Pemphigus foliaceus (PF) is an autoimmune disease primarily driven by autoreactive B cells, characterized by cell detachment between keratinocytes, an acantholytic process that causes skin blisters. This process is caused by autoantibodies against desmoglein 1 (DSG1), a member of the cell-cell adhesion proteins of the desmosomes of keratinocytes (1). Interestingly, PF is uncommon and sporadic in most parts of the globe, exhibiting an incidence as low as one case per million (cpm) (2–5). However, it is an endemic disease in Brazil due to its high incidence in some regions, reaching 25-35 cpm (6), the highest incidence ever reported for an autoimmune disease. Furthermore, an impressive prevalence of up to 3.4% has been reported in Amerindian communities living in the endemic area (7).

Epidemiological studies have shown that most patients live in rural areas or precarious houses, with high exposure to bites from hematophagous insects, considered the most relevant environmental factor associated with the endemicity (8, 9). Some of them are also vectors of parasitic diseases: *L. longipalpis* (leishmaniasis), reduviid (Chagas disease), and simuliid (onchocerciasis), which, besides the disease-causing parasites, also inoculate salivary proteins that could trigger the development of autoreactive antibodies (10). Previous research has shown the presence of non-pathogenic anti-DSG1 antibodies in patients of these diseases (11), and that anti-DSG1 antibodies cross-react with antigens derived from *L. longipalpis* salivary glands, such as LJM17 and LJM11 (12–14), and the peptide maxadilan (15).

On the other hand, several genetic variants strongly increase PF risk, including variants within the major histocompatibility complex (MHC), such as the *human leukocyte antigen* (*HLA*) class I and II genes (16–21). A recent study, also analyzing patients and controls from the endemic area, found that variants in genes related to antiviral responses are associated with higher susceptibility to the disease (22). Altogether, these results suggest that environmental antigens can initiate the autoreactive response of PF in genetically susceptible individuals, which leads to the generation of pathogenic autoantibodies.

Antibody-mediated responses are critical for identifying and protecting against pathogens, toxins, or allergens through specific antigen-binding followed by neutralization, opsonization, complement activation, or stimulation of other immune system cells (23). The failure of self-tolerance mechanisms could lead to a pathogenic response and the development of autoimmune diseases.

Antibodies are encoded by the *immunoglobulin heavy locus* (*IGH*), *lambda locus* (*IGL*), and *kappa locus* (*IGK*) (24, 25) and are secreted by plasma cells. Along with the differentiation of the naïve B cells, these genes undergo somatic recombination of their variable (V), diversity (D), and junction (J) gene segments (26). Upon stimulation, B cells go through somatic hypermutation and class switch recombination after encountering their specific antigens (27, 28). Altogether, these mechanisms generate a great diversity of antibodies capable of recognizing virtually any antigen (29), with the prominent participation of the third complementary-determining region (CDR3), which is primarily responsible for determining the antigen-binding specificity (30).

The rearranged immunoglobulin genes (clonotypes) of anti-DSG1 autoantibodies have been previously described in patients with PF (31, 32). However, the immunoglobulin repertoire of patients and controls from the endemic region has not yet been revealed. Here, we show that the immunoglobulin repertoires differ according to disease status and geography, particularly between individuals from the endemic and non-endemic areas of PF.

## Materials and methods

### Study population

We analyzed 22 unrelated individuals from the PF endemic area in Brazil, divided into four groups: i) non-treated patients (n = 5); ii) patients under immunosuppressive treatment (~30 mg of prednisone/day; n = 5); iii) patients in disease remission (without treatment; n = 6), and iv) healthy controls (n = 6). Another group of v) healthy individuals from outside the endemic area (n = 4) recruited in Curitiba, Brazil, was included for comparison. Individuals in the control group had no reported history of autoimmune disorders, recent infections, or other known medical conditions and did not report to be under any medication. The demographics of the population of this study are shown in **Table 1**.

**Table 1:** Patient diagnostic features and repertoire sequencing.

	Non-treated patients	Patients under treatment	Patients in remission	Controls (endemic region)	Controls (non-endemic region)
Demographic characterization					
Mean Age	35.2	37	44.16	44	43.25
Sex	3F/2M	3F/2M	4F/2M	4F/2M	4F
IGHM repertoire					
Mean raw reads	1,246,283	1,203,399	1,211,195	1,296,708	1,241,521
Mean MIG	2,126.2	2,472.4	1,894.3	1,933.0	17,439.3
Mean clonotypes	1,359.8	1,346.4	1,109.3	1,224.2	12,268.5
IGHG repertoire					
Mean raw reads	2,099,649	1,818,216	1,878,301	1,900,283	1,362,850
Mean MIG	3,939.0	5,584.4	2,550.7	3,904.2	9,571.8
Mean clonotypes	1252.0	1,419.8	452.5	628.0	2,796.0

F, Female. M, Male. MIG, molecular identifier group, i.e., the number of mRNA molecules. Clonotype counts are the number of unique sequences present and sequenced in a sample.

Patients were recruited from hospitals in the endemic area and diagnosed by dermatologists specialized in PF based on immunological tests, histopathology, and immunohistochemistry of skin biopsies. This study was approved by the Human Research Ethics Committee of the Federal University of Parana under protocol number CAAE 02727412.4.0000.0096, according to Brazilian Federal laws and the Declaration of Helsinki.

### Library preparation and sequencing of the immune repertoire

Peripheral blood mononuclear cells (PBMC) were lysed and stored at -80°C in TRizol Reagent (Invitrogen, USA), and total RNA isolation was performed according to the manufacturer’s instructions. We used 100 ng of total RNA for reverse transcription (RT) and library preparation with the SMARTer Human BCR IgG IgM H/K/L Profiling Kit (Takara Bio, USA), which performs a 5’-RACE-like RT and incorporates unique molecular identifiers (UMIs) to each mRNA molecule to facilitate PCR error correction (33). The entire length of the VDJ region and a portion of the constant region of Immunoglobulin Heavy Constant Mu (IGHM) and Immunoglobulin Heavy Constant Gamma (IGHG) transcripts were amplified in two subsequent rounds of PCR. The final product was barcoded with Illumina adapters. We selected 400–900 bp amplicons through purification with magnetic beads (MagSi-NGS). Libraries were analyzed on the Bioanalyzer (Agilent, USA) for quality control and finally pooled at equimolar concentration, denatured, diluted to 12 pM, and sequenced with the MiSeq system (Illumina, USA) using paired-end 2x300 bp protocol (34).

### Sequence analysis and clonotype identification

**Figure 1:**
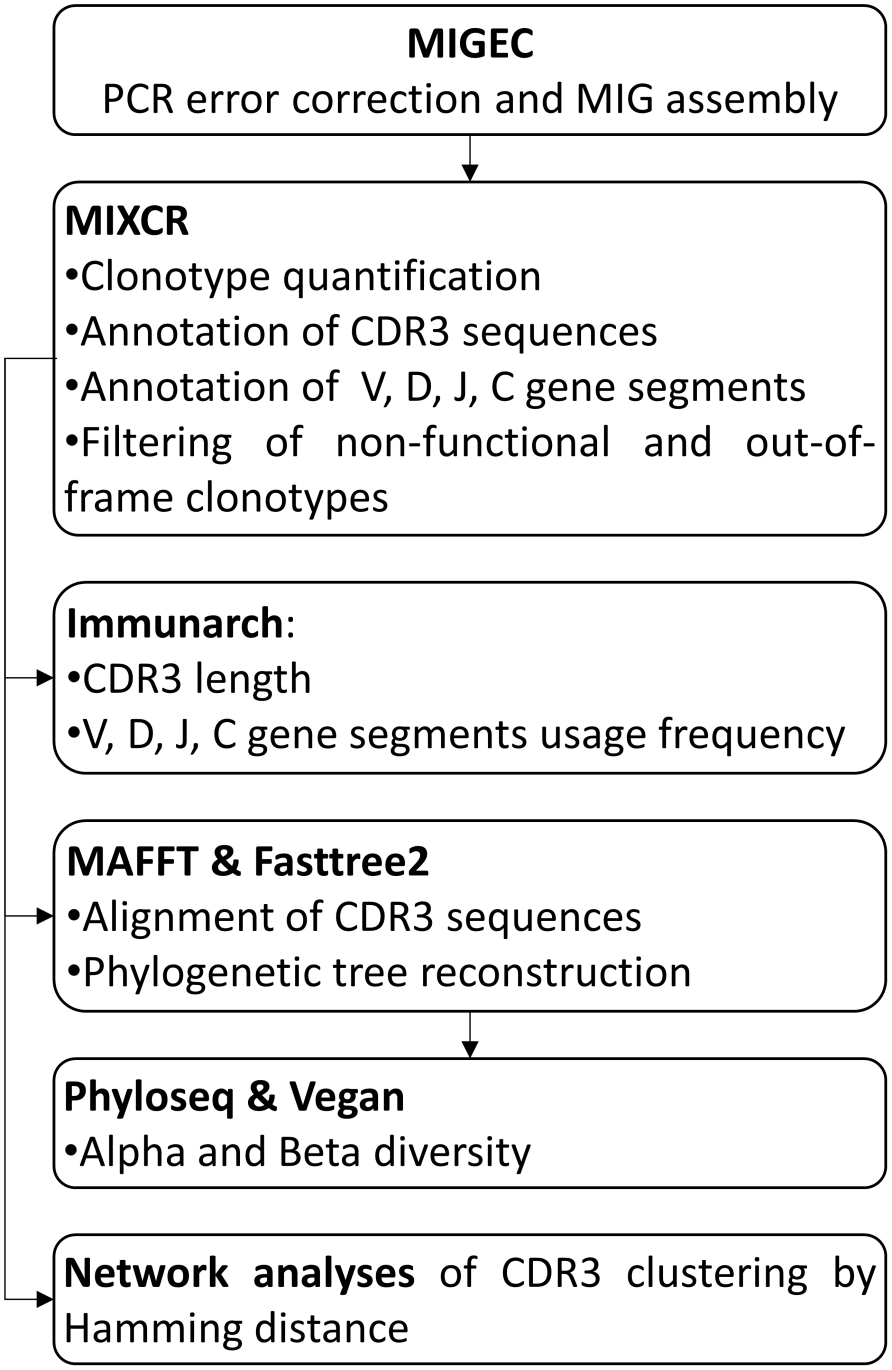
Pipeline for the immune repertoire characterization. MIG: molecular identifier group. CDR3: complementarity determining region 3.

The resulting fastq files were first filtered for high quality and processed for PCR error correction by aggregating UMIs into molecular identifier groups (MIGs) using MIGEC software (33), following the steps shown in **Figure 1**. Annotation of CDR3 sequences and IGHV, IGHD, IGHJ, and IGHC gene segments, as well as clonotype aggregation, quantification, and filtering, were performed with the software MiXCR (35).

### Data analysis

We estimated CDR3 length distribution and the IGHV, IGHJ, and IGHC usage frequency with the R package Immunarch (36). We used the Shapiro–Wilk normality test (37) to analyze the CDR3 length distribution and the Kolmogorov–Smirnov test (38) to compare groups. The Wilcoxon test (39) was applied for the pairwise comparison of usage frequencies for each gene segment between groups. The individual frequencies of the differentially expressed IGHV gene segments were included in a principal component analysis (PCA) to evaluate similarities and possible clustering among samples.

To estimate CDR3 diversity within and among groups, we measured the phylogenetic distance between the CDR3 amino acid sequences of IGHM and IGHG clonotypes by aligning the clonotype sequences using the software MAFFT (v7.471), followed by a phylogenetic tree reconstruction using the software FastTree2 (v2.1.4) (40) with branch length rescaling (gamma option) and generalized time-reversible model (GTR) of nucleotide substitution. We used the R packages phyloseq v1.34.0 (41) and vegan v2.5-7 (42) to estimate alpha and beta diversities, and Faith’s index of phylogenetic distance (PD) (43) and Shannon index (44) to estimate alpha diversity. For beta diversity investigation, we performed a permutational multivariate analysis of variance (PERMANOVA), with 99,999 permutations, on weighted UniFrac distances (45) between clonotypes to identify significant differences among the CDR3 sequences of the two geographic regions (endemic vs. non-endemic). Beta diversity was visualized through a Principal Coordinate Analysis (PCoA). Chemical properties of the clonotypes of each sample were evaluated using the IMGT/V-QUEST tool (46), Change-o software and Alakazam R package (47).

To identify distinctive clonotypes for the endemic area of PF, we evaluated the similarities between the CDR3 sequences of all the samples from the endemic area using network analysis as previously described (48–50). For the network assembly, we considered the CDR3 amino acid sequences as nodes, connected by edges according to their Hamming distance, i.e., the number of amino acid substitutions that differentiate two sequences. Next, we extracted all networks that included clonotypes from at least four different samples from the endemic area connected by a maximum Hamming distance of two. The networks were processed using the igraph R package (51).

## Results

### The subgroups differed in their clonotype frequencies but not in the chemical properties of their clonotypes

We analyzed the immunoglobulin repertoire in three groups of patients, one group of healthy individuals from the PF endemic region in Brazil and another group of healthy individuals from a non-endemic area. We obtained the IGHM and IGHG clonotype repertoire, corresponding to the immunoglobulin heavy chain of IgM and IgG, and studied the whole variable region of the molecule (VDJ exon) and a fragment of the constant region to distinguish the isotypes and subclasses.

**Figure 2:**
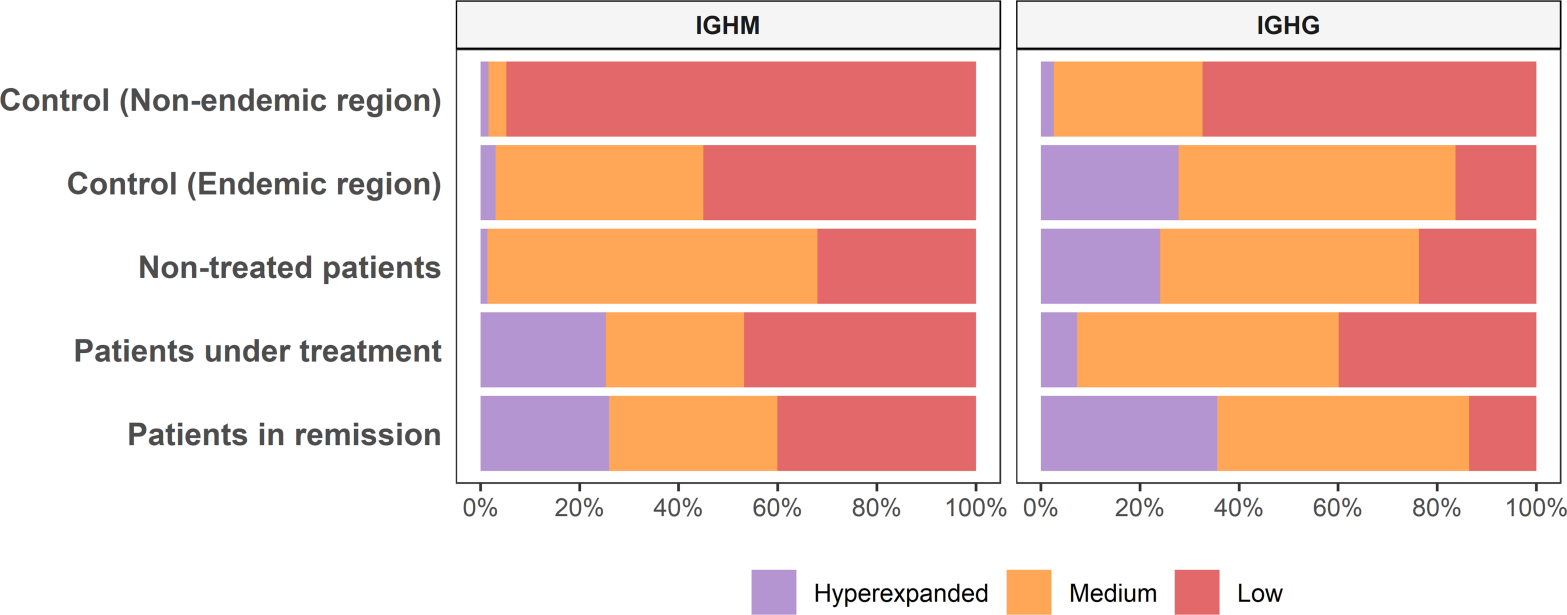
Clonotype abundance among groups. The clonotype frequency proportion differs among groups of samples (Low, < 0.1%; Medium, between 0.1 and 1%; Hyperexpanded, > 1%).

The raw read counts were similar among all groups; however, the mean number of MIGs and clonotypes of the controls from the non-endemic area was up to nine times higher than that of the different patient groups and controls from the endemic area (**Table 1** and **Supplementary Table S1**). We classified the repertoires according to the clonotype frequencies into low (< 0.1%), medium (between 0.1 and 1%), and hyperexpanded (> 1%). Controls from the non-endemic area had a higher proportion of low-frequency clonotypes: 95% and 60% for IGHM and IGHG, respectively. Interestingly, controls from the endemic area presented a significantly larger proportion of medium-frequency and hyperexpanded clonotypes compared to controls from the non-endemic area (*p* < 0.04) (**Figure 2**). Moreover, we compared the proportion of clonotype frequencies between groups and found no statistical differences between all patients and controls from the endemic area. We found significant differences for controls from the non-endemic area compared to patients and controls from the endemic region (**Supplementary Figure S1**). Additionally, the chemical characteristic of the amino acids in the clonotypes did not differ between the samples (**Supplementary Figure S2**).

### Both patients and controls from endemic region present lower clonotype diversity than healthy individuals from the non-endemic area

**Figure 3:**
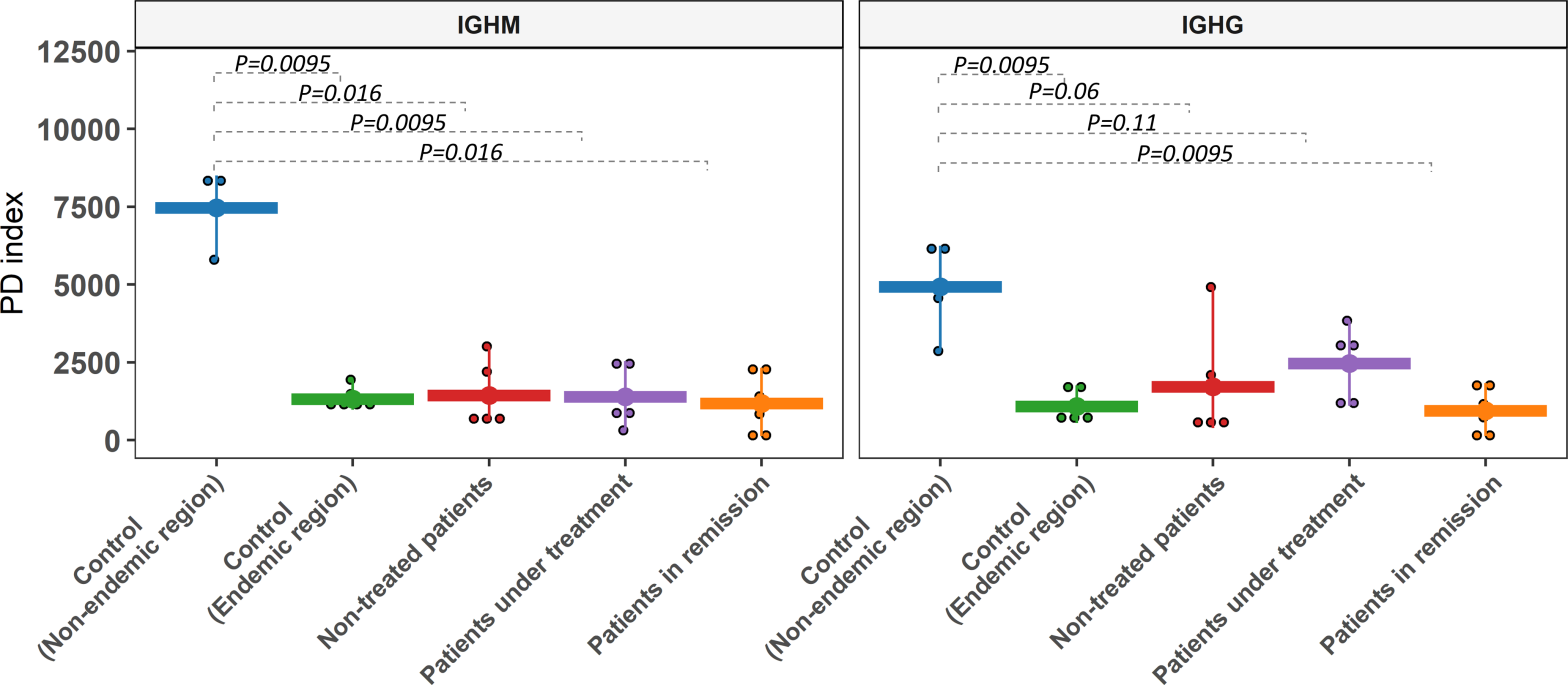
Clonotype diversity of the IGHM and IGHG gene segments. Alpha diversity was assessed with PD (phylogenetic diversity index). Controls from the non-endemic region and controls from the endemic region showed significant differences in most comparisons for both isotypes. Comparisons between endemic samples were non-significant.

We compared the diversity levels between groups through Faith’s phylogenetic diversity index (PD) (43). No significant differences between patient and control groups from the endemic area were observed (*p* > 0.05). However, controls from the non-endemic area exhibited significantly higher diversity than those from the endemic area (*p* < 0.05) for both IgM and IgG (**Figure 3**). This result was confirmed by the Shannon index, which considers the abundance of clonotypes and the evenness of their frequencies (**Supplementary Figure S3**).

**Figure 4:**
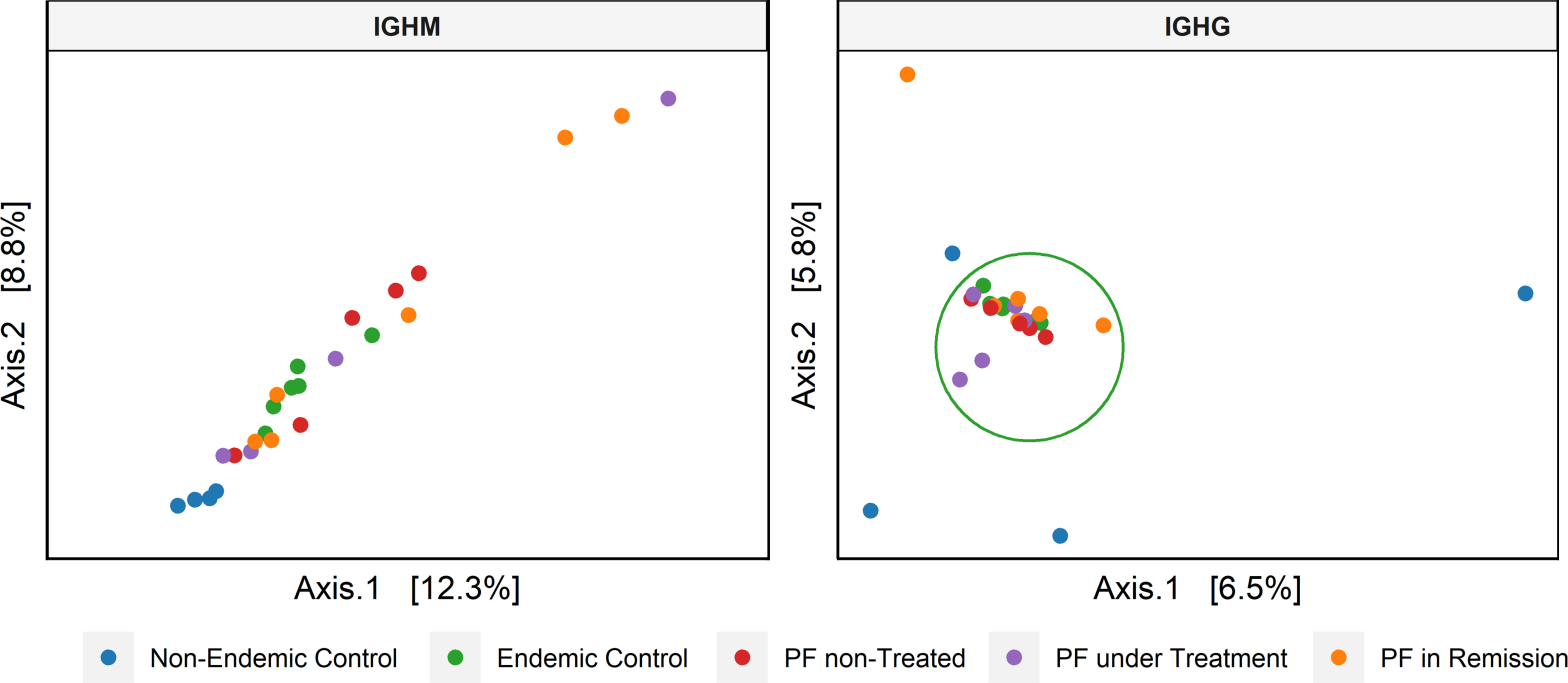
Principal coordinate analysis (PCoA) based on the beta diversity of the CDR3 amino acid sequence. For IGHM, we considered the complete repertoire, showing a tendency of clustering (endemic vs. non-endemic, *p* = 0.11). For IGHG, we only analyzed the clonotypes with medium to high frequencies, showing a significant clustering between regions (endemic vs. non-endemic, *p* = 0.003).

We further evaluated the similarities between CDR3 sequences from the different groups (beta diversity) with the UniFrac distances between the sequences in a phylogenetic tree. We considered medium-frequency and hyperexpanded clonotypes (clonotype frequency > 0.001) for IGHG as they are the most relevant in the repertoire. While for IGHM, we considered all clonotypes due to the restrictively small proportion of high-frequency clonotypes for some groups. From this analysis, we observed only a tendency of separation between non-endemic (blue dots in **Figure 4**) and endemic regions (*p* = 0.11) for IGHM clonotypes. However, a significant difference was observed for IGHG clonotypes between non-endemic (blue dots in **Figure 4**) and endemic regions (*p* = 0.003). There was no differential clustering between controls and patients from the endemic area.

### Differential usage of IGHV5-51 (IGHM) and IGHV3-30 (IGHG) in patients with active disease compared to individuals without disease from the endemic area

**Figure 5:**
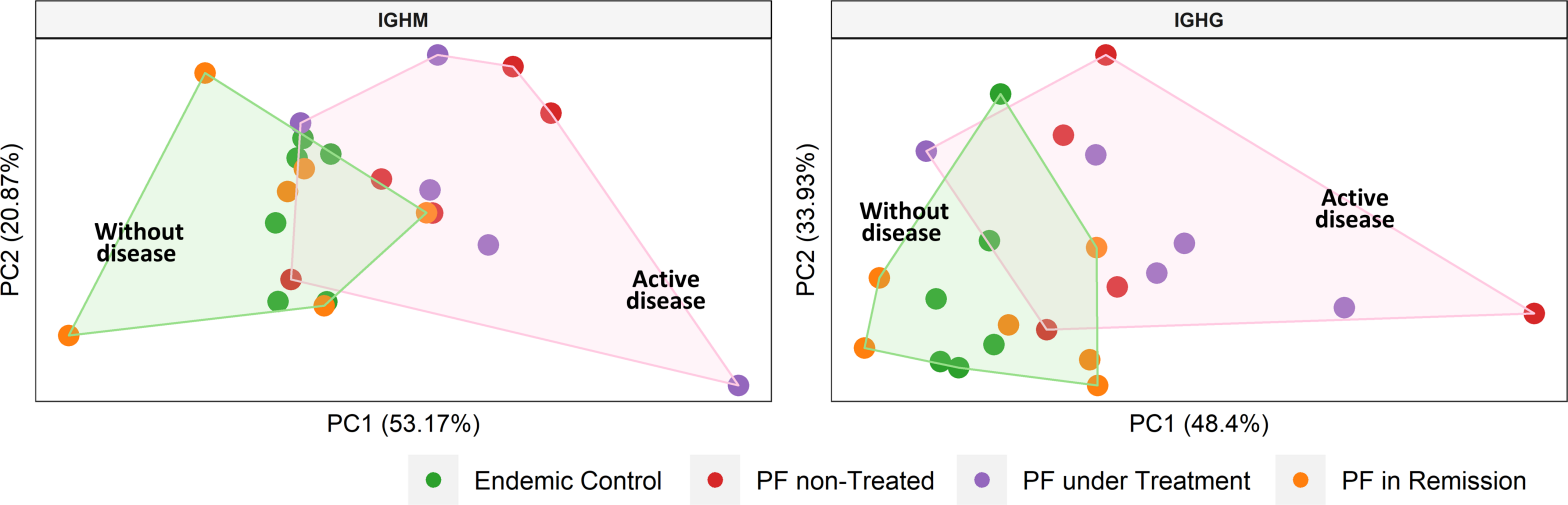
Clustering of samples according to the disease status. We performed a principal component analysis with the frequencies of the IGHV gene segments that were differentially used between groups (see **Table 2**). Shaded areas represent sample groups (pink: individuals with active disease; green: individuals without disease). The group with active disease includes non-treated and under-treatment patients. The group without disease includes controls from the endemic area and patients in remission. PF: Patients with pemphigus foliaceus.

We estimated the usage frequencies of IGHV gene segments in each group (**Supplementary Table S2**) and evaluated if their use differed between groups (**Supplementary Table S3**). PCA separated the two groups i.e., with active disease (non-treated PF patients and PF patients under treatment) and without the disease (controls from the endemic region and PF patients in remission) for both IGHM and IGHG isotypes (**Supplementary Figure S4).** Based on these results, we evaluated the IGHV gene segments differentially used between groups for both IGHM and IGHG isotypes (**Table 2**), which one more time separated the groups by disease status (**Figure 5**). The most frequent segments in patients with active disease were IGHV5-51 (IGHM) and IGHV3-30 (IGHG), while IGHV1-69 (IGHM) and IGHV3-23 (IGHG) were the most frequent in individuals without the disease (*p* < 0.05; **Table 2**). We found neither significant differential usage for most IGHJ gene segments (**Supplementary Figure S4A**) nor differences in the IGHG isotype frequencies (**Supplementary Figure S4B**). A PCA on the IGHV gene segments indicated significantly different frequencies between individuals with active disease (non-treated and under-treatment patients) and individuals without the disease (patients in remission and controls), and found a separation between them (**Figure 5**).

**Table 2:** Comparison of the IGHV usage in individuals with active pemphigus foliaceus and without disease.

Isotype	Gene segment	Frequency		*p*-value
		Without disease	Active disease	
IGHM	IGHV1-69	0.076	0.052	0.036
	IGHV2-5	0.008	0.014	0.038
	IGHV3-73	0.006	0.011	0.032
	IGHV5-51	0.026	0.037	0.002
IGHG	IGHV1-58	0.001	0.002	0.021
	IGHV3-23	0.098	0.080	0.017
	IGHV3-30	0.097	0.115	0.043

Active disease = patients with active PF, with (n=5) and without treatment (n=5); without disease = healthy individuals from the endemic area (n=6) and patients in remission (n=6).

### IGHG clonotype of PF patients without immunosuppressive treatment have longer CDR3 amino acid sequences

**Figure 6:**
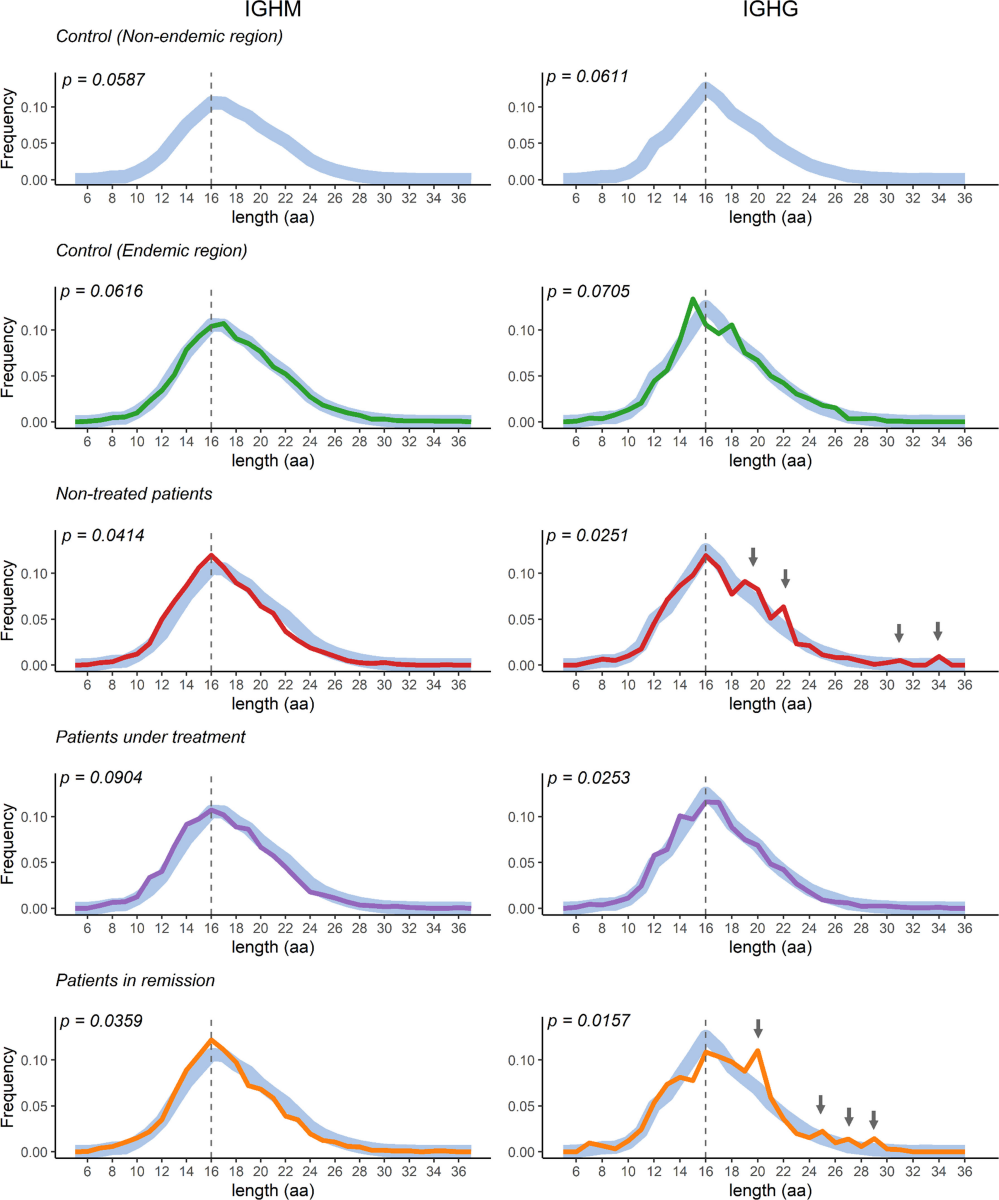
CDR3 length distribution in the study population groups. The curves represent the distribution of the CDR3 region’s length. Control samples from the non-endemic region (represented as thick light-blue curves) were compared to samples from the endemic region. Deviations from the Gaussian distribution were only observed in patients (*p* < 0.05, Shapiro-Wilk normality test). The vertical dotted line represents the CDR3 median length. aa = amino acid.

For an unperturbed repertoire, it is expected that the CDR3 distribution follows a normal Gaussian distribution (52). However, a skewed or deviation from normality could indicate an over-abundance of some clones, perhaps due to stimulation from specific antigens. We observed a normal distribution of IGHM and IGHG CDR3 lengths (*p* > 0.05, Shapiro-Wilk normality test) in controls, while the distribution deviated from normality in all patient groups, except the IGHM distribution in patients under treatment (**Figure 6**). We found no statistical differences between the CDR3 length distribution of patients compared to controls of both the endemic and non-endemic regions (*p* > 0.05, **Supplementary Table S4**). However, we observed an increased frequency of IGHG clonotypes with longer CDR3 sequences in patients without immunosuppressive treatment and patients in remission (indicated by arrows in **Figure 6**).

### Network analysis of CDR3 sequences reveals clonotype clusters that might be implicated in pemphigus pathogenesis

**Figure 7:**
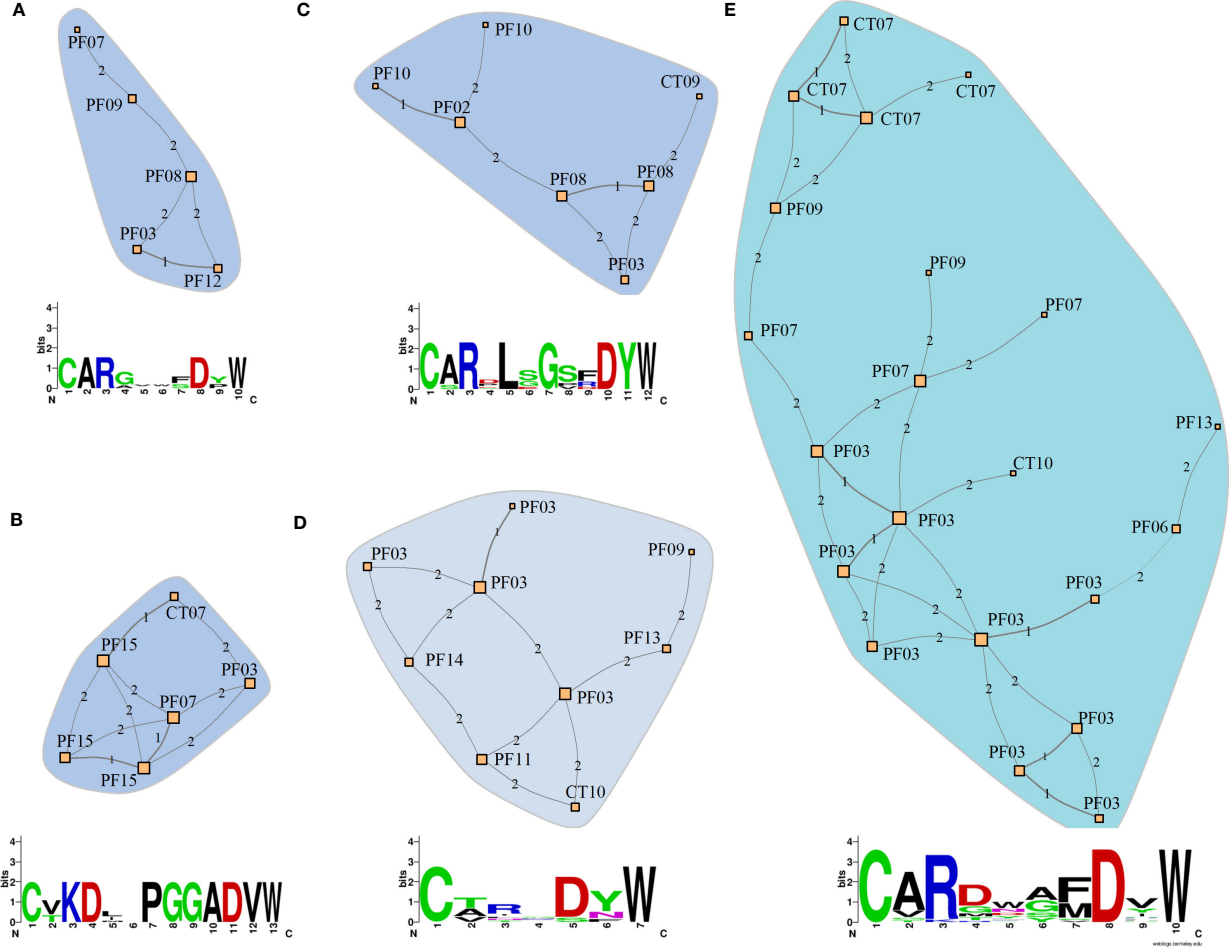
Similarity networks among clonotypes of patients and controls from the endemic area. **(A)** Network consisting solely of clonotypes from patients (PF). **(B-E)** Networks consisting predominantly of clonotypes from patients with a few from controls (CT) from the endemic area (Details of the network composition and clonotype sequences are shown in **Supplementary Table. S5**). The networks were constructed based on CDR3 amino acid sequences of IGHG clonotypes. The nodes represent unique clonotype sequences and are labeled with the sample IDs. Each clonotype differs from the adjacent clonotype by the quantity of amino acid residues indicated by the numbers between nodes (Hamming distances). The amino acid consensus sequences indicate the similarity between clonotype sequences in each network. Letter heights indicate the conservation in each position, with the most frequent amino acid placed on top.

Similarities between the clonotype sequences of patients could reflect similar binding properties in PF-related autoantibodies. To estimate these similarities, we compared the CDR3 sequences from clonotypes of patients and controls from the endemic region. We considered a cluster relevant for PF if it connected clonotypes of at least four distinct patients (**Figure 7**), meaning that at least four individuals have similar clonotype sequences in each network. One of these networks consists of clonotypes exclusively found in patients (**Figure 7A**), while the other four networks include clonotypes found primarily in patients (**Figures 7B-D**). Below each network, we present the consensus sequence representing the similarity among the CDR3 sequences of the clonotypes. No network comprising clonotypes exclusively from controls could be identified. The CDR3 sequences and their associated IGHV, IGHJ, and IGHG segments are available in **Supplementary Table S5**.

## Discussion

Here, we present the first analysis of the IGHM and IGHG immune repertoire in patients with endemic PF, delivering the complete landscape of IgM and IgG in the peripheral blood of patients and healthy controls. Because PF is the only known autoimmune disease that is also endemic, comparing healthy controls from the endemic and non-endemic areas provides new insights into the still unknown environmental triggers, such as pathogens and antigens related to living conditions (53). In parallel, comparing healthy individuals with patients helps distinguish pathogenic from non-pathogenic characteristics in the B-cell repertoire.

We observed that individuals from the endemic area exhibited a remarkably lower clonotype diversity than controls from outside this area. Different parameters, such as MIG and clonotype counts, frequencies, and diversity indexes, highlight these differences. Our study demonstrated a small repertoire diversity in all individuals from the endemic region, regardless of whether they were healthy or PF patients. By contrast, we showed that repertoires in most individuals from the non-endemic area consist of low-frequency clonotypes, consistent with a low selective constraint in this environment. In sharp contrast, medium frequency and hyperexpanded clonotypes are more frequent in patients and controls from the endemic area, indicating intense and persistent environmental immunostimulation.

We also observed that patients with non-treated PF exhibited more medium-frequency IGHM clonotypes, which could result from an early response against recent, new antigens exposed in the skin lesions, as suggested by Grando et al. (54). The high frequency of IGHG hyperexpanded clonotypes in patients is characteristic of an ongoing intense immune response against specific antigens (55). Besides, the reduction of hyperexpanded clonotypes in treated patients is consistent with the immunosuppression caused by the treatment. Overall, our results indicate that the immune repertoire is shaped differently in the PF endemic area, possibly determined by an environmental factor driving the expansion of specific clonotypes while decreasing the overall clonotype diversity. These observations, taken together with the previously mentioned studies (12, 13, 22), which had different hypotheses and methodological approaches, reinforce the idea that environmental factors overstimulate the immunological repertoire and trigger PF in the endemic region.

In the PCoA based on beta-diversity, the separate clustering of individuals from endemic and non-endemic areas is due to the greater similarity between CDR3 sequences in individuals from the endemic area, which results in a lower UniFrac distance between their clonotypes. This result indicates similar selective constraints and stimulation by the same environmental factor during immune repertoire development (56). Past studies support this observation by showing that patients and healthy individuals from the indigenous community of Limão Verde, located within the Brazilian endemic PF region, exhibited high anti-DSG1 IgG levels, possibly due to a constant exposition to an antigen that elicits immune responses generating autoantibodies. This immune response can culminate in pathological autoimmunity in genetically susceptible individuals. By contrast, anti-DSG1 levels are lower in healthy individuals from other Brazilian localities and other countries (57).

Studying IGHV usage is crucial because these gene segments encode the immunoglobulin CDR1 and CDR2 regions, which, together with CDR3 (58), are critical for antigen binding and define antibody-antigen affinity (59). IGHV utilization during immunoglobulin rearrangement is not entirely random (60), and some IGHV segments are differentially used in the repertoire of healthy individuals, while others are overrepresented in some diseases (61). The PCA based on the IGHV gene segment usage separated individuals with and without active PF, indicating differential gene usage among these two groups. Some of the gene segments that exhibited higher frequency in patients with active disease, such as IGHV2-5, V3-73, and V3-30, have been previously identified in anti-DSG autoantibodies from patients with PF and PV (31, 32). It is important to note that our results show a shift in the complete repertoire of patients with active PF compared to individuals without disease. However, our analysis does not account only for the pathogenic anti-DSG1 antibodies, as visible by the fact that IGHV3-23 gene segment has higher expression in our control group, but has also been found previously in anti-DSG1 clonotypes. This differential IGHV usage in individuals with skin lesions may be implicated in developing pathogenic antibodies, which could target self-antigens in susceptible individuals, not only DSG1 but also other skin molecules.

An interesting feature in immunoglobulin repertoire studies is the analysis of the length of the CDR3 region, as it can indicate repertoire unbalance (52). We found that patients presented a deviation from normality in their distribution of CDR3 length, with an overrepresentation of clonotypes with longer CDR3 sequences. Similar findings were observed in other autoimmune diseases, such as immunoglobulin A nephropathy, Crohn’s disease, and systemic lupus erythematosus (62, 63). Longer CDR3 sequences have been associated with more flexible and more polyreactive antibodies (64) and thus could be more prone to autoreactivity (65).

We analyzed the similarity among the clonotypes in patients with PF to identify possible CDR3 sequences that could potentially lead to the discovery of environmental antigens or additional self-antigens involved in this disease. Although previous studies focusing on anti-DSG1 antibodies indicated no convergence in their clonotype sequences (31, 32), we aimed to uncover distinguishing sequences in the complete PF repertoire using similarity networks of clonotypes based on the Hamming distances of the CDR3 sequences. This method computes the number of mismatched amino acids considering only those sequences of equal length. The underlying assumption is that both sequence and length influence the binding properties of the antibodies to the antigens. Thus, closely related sequences may imply comparable epitope binding properties and antigen specificity (66). Accordingly, the CDR3 sequences clustering in the network analysis could bind the same autoantigen or environmental antigen. This applies especially to the PF-relevant antibodies in network A, composed exclusively of clonotypes from patients. It is important to acknowledge the limitation that the epitopes bound by these clonotypes are unknown. However, we provide a comprehensive overview of the clonotypes present in PF patients of the endemic region of Brazil, which may facilitate the recognition of relevant clonotypes in future research efforts and elucidate disease-associated mechanisms by testing functional hypotheses.

Previous studies have shown that the IgG1 isotype is primarily present in healthy individuals and patients before disease onset and that most of the pathogenic anti-DSG1 antibodies in patients with PF are IgG4 (67). For this reason, we wanted to evaluate if there was also a change of IGHG gene segment expression in the entire repertoire. However, our library preparation method could not distinguish between IGHG3 and IGHG4 gene segments. Nevertheless, our observation that IGHG segments (IGHG1, IGHG2, and IGHG3/4) presented similar distribution among patients and controls (**Supplementary Figure S4B**) indicates no differential switching from IgG1 to IgG4 in patients when considering the whole repertoire. Another limitation of the present study is the small number of subjects in each group due to the difficulty in accruing samples from such an uncommon disease and patients in specific testing conditions. However, the individuals were carefully chosen to ensure that they were representative of the target population. For this reason, we believe our results are reliable and provide valuable insights into the repertoire of individuals living in the endemic area of PF in Brazil.

## Conclusions

We comprehensively characterized the immunoglobulin repertoires of patients with endemic pemphigus foliaceus and compared them to those of controls from both endemic and non-endemic Brazilian areas. The immunoglobulin repertoire is profoundly distinct in the endemic area, as we observed lower clonotype counts, lower diversity, and a higher proportion of medium and hyperexpanded clonotypes in both patients and controls. The significant differences in the immunoglobulin repertoire of controls from the non-endemic and endemic areas and the similarity between patients and controls from the endemic area likely result from the antigen that triggers endemic PF. This antigen is characteristic of the endemic region and could be derived from a pathogen or a hematophagous insect’s salivary protein. The immune response to this antigen culminates in a pathogenic autoimmune response only in genetically susceptible individuals.

Patients with PF exhibited a deviation from the normal distribution of CDR3 lengths and a higher frequency of longer CDR3 sequences, a characteristic previously associated with autoreactive antibodies. Additionally, patients from the endemic area with active disease exhibited a differential usage of IGHV segments. Specifically, IGHV3-30 was more frequent in patients with lesions. We identified one cluster of clonotypes that could belong to antibodies involved in PF pathogenesis. Further investigation of this cluster and its consensus sequence might be worthwhile in future studies focusing on identifying the environmental trigger, especially under the recent suggestion that a virus might also trigger PF. In addition, these clusters might inform about self-antigens besides DSG1 and might contribute to identifying targets for therapeutical B cell depletion.

## Data availability statement

The original contributions presented in the study are included in the **Supplementary Material**. Raw data is deposited in The Sequence Read Archive (SRA) under the accession number PRJNA992407. Further inquiries can be directed to the corresponding authors.

## Ethics statement

The studies involving human participants were reviewed and approved by Human Research Ethics Committee of the Federal University of Parana under protocol number CAAE 02727412.4.0000.0096. The patients/participants provided their written informed consent to participate in this study.

## Author contributions

VCS, DGA, and AF conceived and designed the study. DGA, MLPE and HB financed the research. GAC, CMC, DM, and TDJF collected the samples. VCS, DGA, and AF performed library preparation and sequencing. VCS, MO, AF, FO, AK, HB analyzed the data. VCS and DGA drafted the manuscript. All authors contributed to the article and approved the submitted version.
