# Supplementary material for: The landscape of the immunoglobulin repertoire in endemic pemphigus foliaceus
Source: Front Immunol. 2023 Jul 28;14:1189251. doi: 10.3389/fimmu.2023.1189251 (PMC10421657; doi:10.3389/fimmu.2023.1189251)
Supplement: Supplementary file 1 [file DataSheet_1.pdf]

This section contains the supplementary figures supporting the results found in the manuscript

"The landscape of the immunoglobulin repertoire in endemic pemphigus foliaceus"

Calonga-Solís V, Olbrich M, Ott F, Adelman Cipolla G, Malheiros D, Künstner A, Farias TDJ, Camargo CM, Petzl-Erler ML, Busch H, Fähnrich A and Augusto DG (2023) *Front. Immunol*.

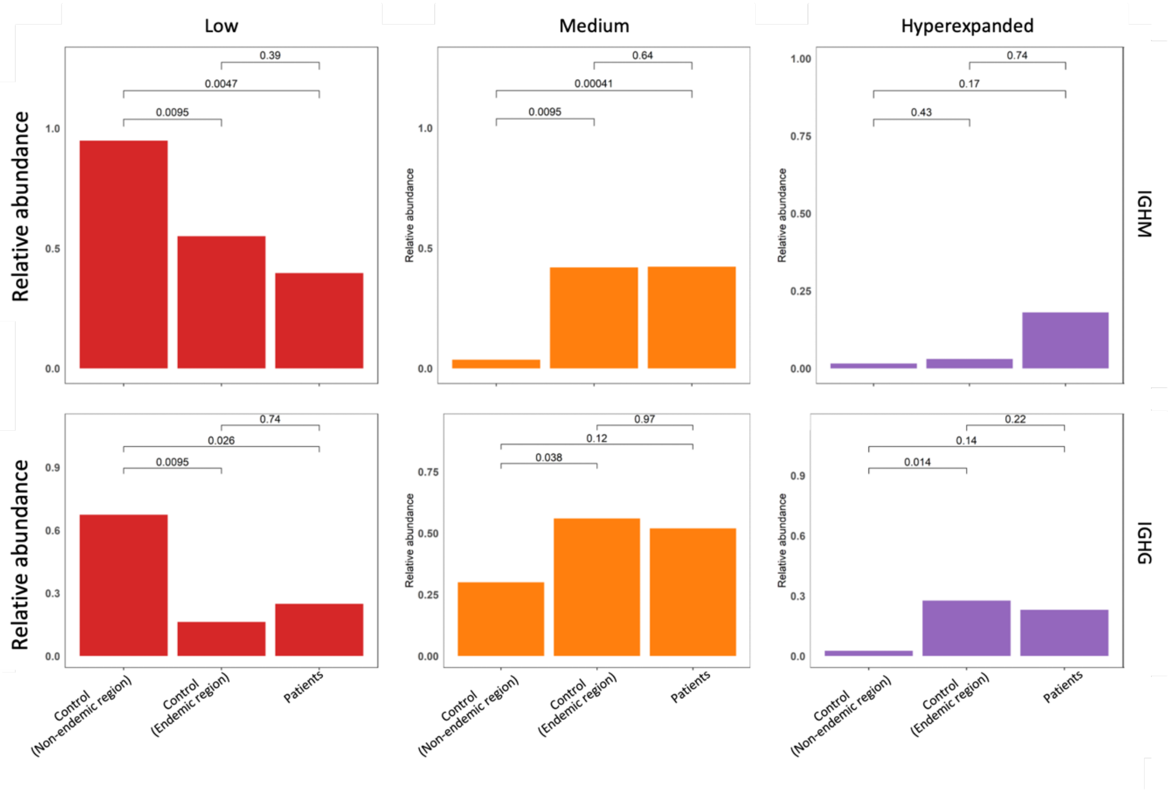

Supplementary Figure S1: Comparison of clonotype frequency proportions between controls of the non-endemic area, of the endemic area, and patients. The statistical differences between pair of groups were evaluated with a Wilcoxon test. Clonotypes are classified according to their frequencies in Low, < 0.1%; Medium, between 0.1 and 1%; and Hyperexpanded, > 1%.

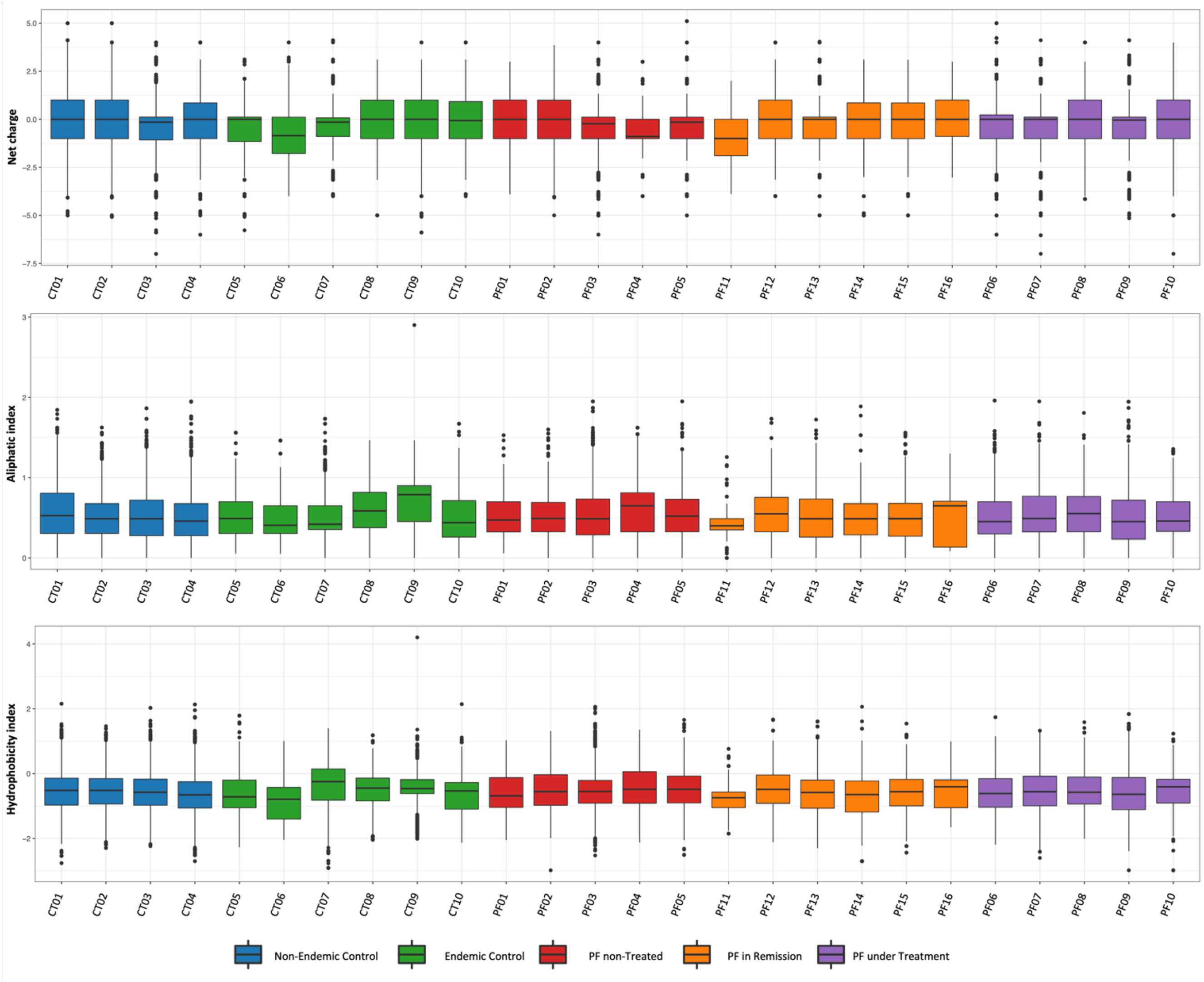

Supplementary Figure S2. Clonotype chemical amino acid properties did not differ among samples. Each dot represents the average scores for the amino acids in one clonotype.

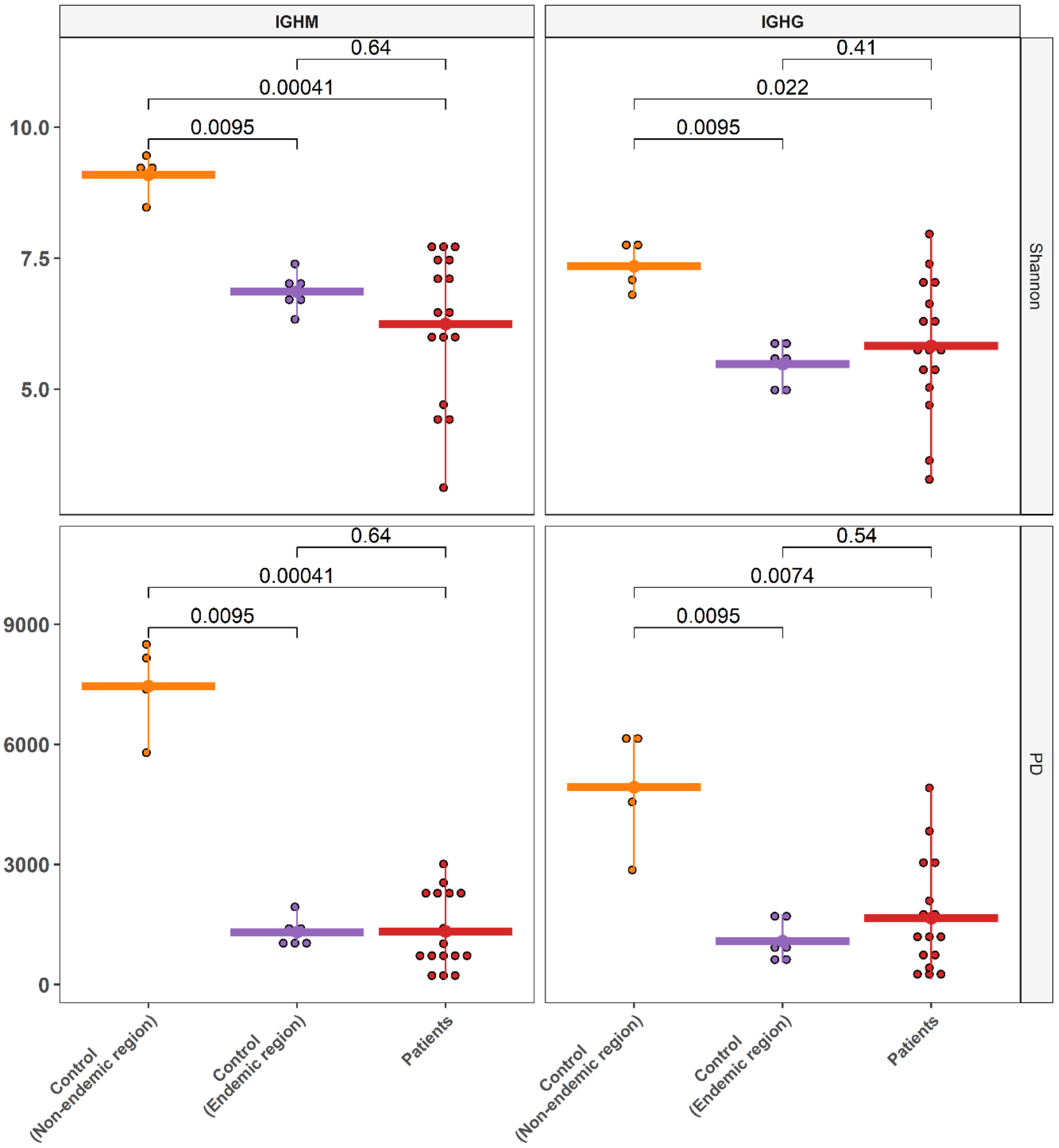

Supplementary Figure S3: Alpha diversity values for IGHM and IGHG clonotypes of the controls from the non-endemic area, controls from the endemic area and patients. Each dot represents the value of Shannon and Faith Phylogenetic Diversity indices in each sample in their respective group. The horizontal bars indicate the mean diversity value of the groups, and the vertical bars the distribution in each group. The statistical differences between pair of groups were evaluated with a Wilcoxon test.

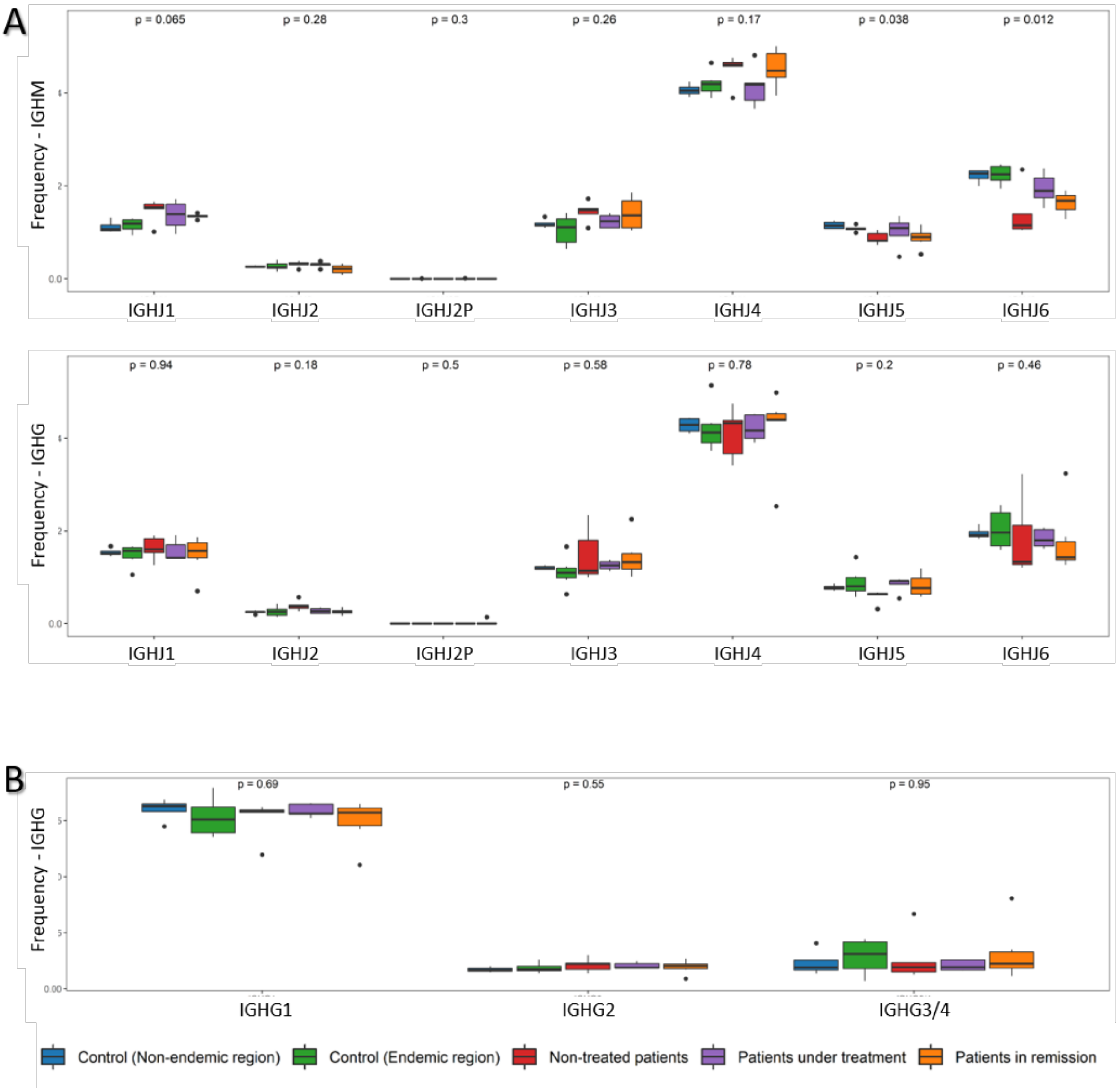

Supplementary Figure S4: (A) Boxplot of the frequency of usage of IGHJ gene segments in IGHM and IGHG clonotypes and (B) of IGHG isotypes in each group. Statistical comparisons were performed using Kruskal-Wallis for each gene segment between all groups.

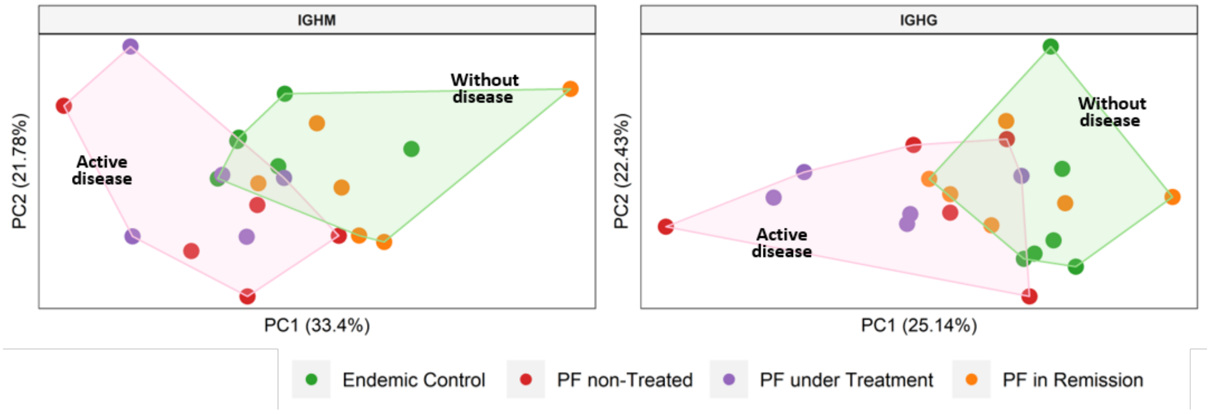

Supplementary Figure S4: Clustering of samples according to the disease status. We performed a principal component analysis with the frequencies of the IGHV gene segments that were differentially used between pair of groups (see Suppl. Table S3). Shaded areas represent sample groups (pink: individuals with active disease; green: individuals without disease). The group with active disease includes non-treated and under-treatment patients. The group without disease includes controls from the endemic area and patients in remission. PF: Patients with pemphigus foliaceus
